# Usability of the Swedish Accessible Electronic Health Record: Qualitative Survey Study

**DOI:** 10.2196/37192

**Published:** 2022-06-23

**Authors:** Maria Hägglund, Isabella Scandurra

**Affiliations:** 1 Healthcare Sciences and e-Health Department of Women's and Children's Health Uppsala University Uppsala Sweden; 2 Centre of Empirical Research on Information Systems School of Business Örebro University Örebro Sweden

**Keywords:** usability, evaluation, patient-accessible electronic health records, open notes, patient portals, mobile phone

## Abstract

**Background:**

Patient portals are increasingly being implemented worldwide to ensure that patients have timely access to their health data, including patients’ access to their electronic health records. In Sweden, the e-service Journalen is a national patient-accessible electronic health record (PAEHR), accessible on the web through the national patient portal. User characteristics and perceived benefits of using a PAEHR will influence behavioral intentions to use and adoption; however, poor usability, which increases effort expectancy, may have a negative impact. Therefore, it is of interest to further explore how users of the PAEHR Journalen perceive its usability and usefulness.

**Objective:**

On the basis of the analysis of the survey respondents’ experiences of the usability of the Swedish PAEHR, this study aimed to identify specific usability problems that may need to be addressed in the future.

**Methods:**

A survey study was conducted to elicit opinions and experiences of patients using Journalen. Data were collected from June to October 2016. The questionnaire included a free-text question regarding the usability of the system, and the responses were analyzed using content analysis with a sociotechnical framework as guidance when grouping identified usability issues.

**Results:**

During the survey period, 423,141 users logged into Journalen, of whom 2587 (0.61%) completed the survey (unique users who logged in; response rate 0.61%). Of the 2587 respondents, 186 (7.19%) provided free-text comments on the usability questions. The analysis resulted in 19 categories, which could be grouped under 7 of the 8 dimensions in the sociotechnical framework of Sittig and Singh. The most frequently mentioned problems were related to regional access limitations, structure and navigation of the patient portal, and language and understanding.

**Conclusions:**

Although the survey respondents, who were also end users of the PAEHR Journalen, were overall satisfied with its usability, they also experienced important challenges when accessing their records. For all patients to be able to reap the benefits of record access, it is essential to understand both the usability challenges they encounter and, more broadly, how policies, regulations, and technical implementation decisions affect the usefulness of record access. The results presented here are specific to the Swedish PAEHR Journalen but also provide important insights into how design and implementation of record access can be improved in any context.

## Introduction

### Background

Patient portals are increasingly being implemented worldwide to ensure that patients have timely access to their health data and a means of communicating with health care professionals and managing their health care [1]. Although they were originally mostly for administration, patients are now gaining access to not only test results but also their full electronic health record (EHR), including notes written by health care professionals.

Patient-accessible EHRs (PAEHRs) have been or are being implemented in many countries, such as Finland, France, Norway, Australia, Denmark, Canada, the United Kingdom, and Sweden [2]. In some countries, these are local implementations at a specific hospital or region, whereas others have national solutions [3]. Differences in strategies and approaches have affected uptake and impact, and in several countries, the implementation progress has been slow because of legal constraints [4,5] and concerns about, for example, security and privacy among medical professionals [6-8].

In the United States, the OpenNotes initiative for providing patients access to their EHR began as a pilot and evaluation project that included 105 volunteer primary care physicians and their 19,000 patients [9,10]. The initiative started in 2010 and has since spread throughout the United States [11]. As of April 2021, a federal rule requires US health care providers to allow patients access to all health information in their EHR [12,13].

In Sweden, *Journalen* is a national PAEHR that is accessible via the web through the national patient portal 1177.se [14]. The PAEHR service accesses EHR information from different EHR systems used throughout Swedish health care organizations through a national health information exchange platform [15,16]. Therefore, patients have one access point for all their health record information [14]. Since the first Swedish region began providing its inhabitants web-based access to their health records in 2012, all other regions have connected to the national infrastructure and the PAEHR Journalen, with the last connection in March 2018. Different regions have also made different choices regarding how much of their information would be made available to patients [14,17].

A challenge that is frequently described internationally is the low adoption rate of patient portals and PAEHRs. This is often attributed to either perceived low usefulness or poor usability in combination with low eHealth literacy among users [18]. Therefore, it is of interest to explore how users of PAEHR Journalen perceive its usability and usefulness.

In this study, we analyzed data on usability issues from a national survey conducted among patients who used the PAEHR Journalen. A first analysis of the main results from the survey was published in 2018 [17] and contained an overview of the full survey. Usability was assessed in the survey using the System Usability Scale (SUS) [19,20], and the results of the SUS analysis indicated that the PAEHR was rated fairly highly by the respondents (81 on the SUS scale) [21]. However, as such, the SUS scale does not give any indication of what types of usability problems end users experience or the severity. Therefore, in this study, we analyzed qualitative free-text comments related to the usability of the PAEHR from the same national survey.

### Objectives

On the basis of the analysis of the survey respondents’ experiences of the usability of the Swedish PAEHR, this study aimed to identify specific usability problems that may need to be addressed in the future. Regardless of whether the problems were frequent or disturbed *all* users, or whether the usability problems were severe or important to *some* users, the aim was to cover different aspects and indicate where future efforts need to be put.

## Methods

### Overview

A survey study was conducted to elicit the opinions and experiences of patients using Journalen. Participants were recruited through the national PAEHR Journalen. When patients logged into Journalen, they received a request for voluntary survey participation together with information about the study.

At the time of data collection (June to October 2016), not all regions were providing patients access to their records through Journalen, and among those who did, the level of transparency varied [17]. Of 22 health care providers (21 regions and 1 private health care provider), 18 (82%) gave patients web-based access to notes in the record, whereas only 8 (36%) gave access to laboratory results and 7 (32%) to immunizations [17]. Notes from psychiatric care were shared by only 2 (9%) health care providers, a number that has increased rapidly since [22].

### Data Collection

A questionnaire was designed covering different topic areas with 24 questions, in Swedish (see the full questionnaire in the study by Moll et al [17]), including questions regarding the usability of the system using SUS [21], followed by a free-text comment where the respondents could add anything they wanted regarding the usability of the system. Thus, the resulting free-text comments were the material for this study.

The usability and technical functionality of the electronic questionnaire were not tested before fielding the questionnaire. However, participants received information about whom to contact in case of technical issues.

The collected data were managed by the national eHealth service provider Inera AB in accordance with the security requirements presented in the ethical application and approved by the Regional Ethical Review Board. The survey data were stored in the same database system as the PAEHR, indicating that the collected data, including the patient ID, had the same security protection as all patient information handled in the PAEHR. The patient ID was stored during the collection period to ensure that patients did not leave duplicate responses. When the collection period was completed, the patient ID was removed, and all stored information was anonymized. The anonymized data set was exported to the researchers for analysis.

### Data Analysis

Overall, 2587 patients from 21 county councils completed the survey. The number of respondents for each county council or region varied. Only completed questionnaires were included for analysis, as the answers were stored in the database only when the respondent chose to submit them on the last page.

In this study, we focused on free-text answers related to the usability of Journalen. Free-text answers regarding usability were analyzed through inductive content analysis, as proposed by Graneheim and Lundman [23]. Questions regarding demography and the perceived usefulness of the overall survey are also presented.

In total, 186 respondents voluntarily provided free-text answers to the question about Journalen’s usability. The answers were read independently by both authors (MH and IS), and *meaning-bearing units* or *meaning units* were identified and coded according to their content [23]. Graneheim and Lundman [23] define *meaning units* as “words, sentences or paragraphs containing aspects related to each other through their content and context.” Most comments were short and equaled one *meaning-bearing unit*; however, some contained more information and were represented by ≥2 *meaning-bearing units*. Therefore, the total number of coded *meaning-bearing units* was >186.

The analysis began with each author performing a traditional deductive content analysis [23], independently categorizing the identified *meaning-bearing units* into categories. However, we quickly saw that the content of the comments went beyond traditional usability issues and matched the 8 dimensions of the sociotechnical framework by Sittig and Singh [24]. Therefore, we decided to use this framework to group the categories that emerged. The eight dimensions were (1) hardware and software computing infrastructure; (2) clinical content; (3) human-computer interface; (4) people; (5) workflow and communication; (6) internal organizational policies, procedures, and culture; (7) external rules, regulations, and pressures; and (8) system measurement and monitoring.

### Ethics Approval

Data were collected from June to October 2016 after ethics approval of the research was granted by the Regional Ethical Review Board in Uppsala, Sweden (EPN 2016/129).

## Results

### Overview

During the survey period, 423,141 unique users logged in to Journalen, of whom 2587 (0.61%) patients completed the survey. Of all the respondents, 62.97% (1629/2587) identified as women and 30.85% (798/2587) as men; 0.39% (10/2587) of the respondents chose *other*, and 5.8% (150/2587) did not answer this question. According to use statistics provided by Inera AB, the company providing Journalen and the national patient portal [25], this reflects the gender distribution of the users in general (in 2016: 60% women and 40% men). Of all respondents, 39.81% (1030/2587) stated that they were working or had been working within health care, and 54.54% (1411/2587) stated that they had no professional relation to health care; 5.64% (146/2587) of respondents did not answer this question. Respondents to this survey had a higher education level than the general population [17]. Among our respondents, 60.57% (1487/2455) had higher education compared with 42% of the general population [26]. Whether this is because users of Journalen are well educated or whether this is a subgroup of users who are more inclined to answer a survey is not known.

In summary, the survey results regarding user characteristics at the national level indicated that most respondents were women and that most had studied at least 3 years of higher education. In addition, the results indicate that many users of Journalen experienced both being patients and working as health care professionals.

In the overview of the survey results by Moll et al [17], details of the respondents’ views on the usefulness and benefits of accessing their health records on the web are presented in more detail. Overall, the patients who answered the survey were positive toward Journalen. Respondents were asked to rate on a 5-point Likert scale the extent to which they agreed to the following more general statements: “I think that access to one’s medical records online is generally a good reform” and “I think that access to Journalen is good for me.”

Of all the respondents, >96% (2454/2541, 96.58%, and 2455/2528, 97.11%) for the respective questions showed a positive attitude toward Journalen (completely or partly agree). However, a positive attitude toward having access to one’s health records does not say much about the usability of the system; therefore, we now present an analysis of the free-text comments related to the usability of the Swedish PAEHR.

### Qualitative Analysis of Free-Text Answers

#### Overview

Analyzing free-text comments qualitatively adds to a deeper understanding of the usability issues patients experience when using a system, which, in this case, is Journalen. Of the 2587 respondents, 186 (7.19%) provided free-text comments on the usability questions. Most comments were short; however, some contained more information and were coded into several categories. A total of 9 comments did not include any useful information and were excluded from the presentation of the results.

**Figure 1:**
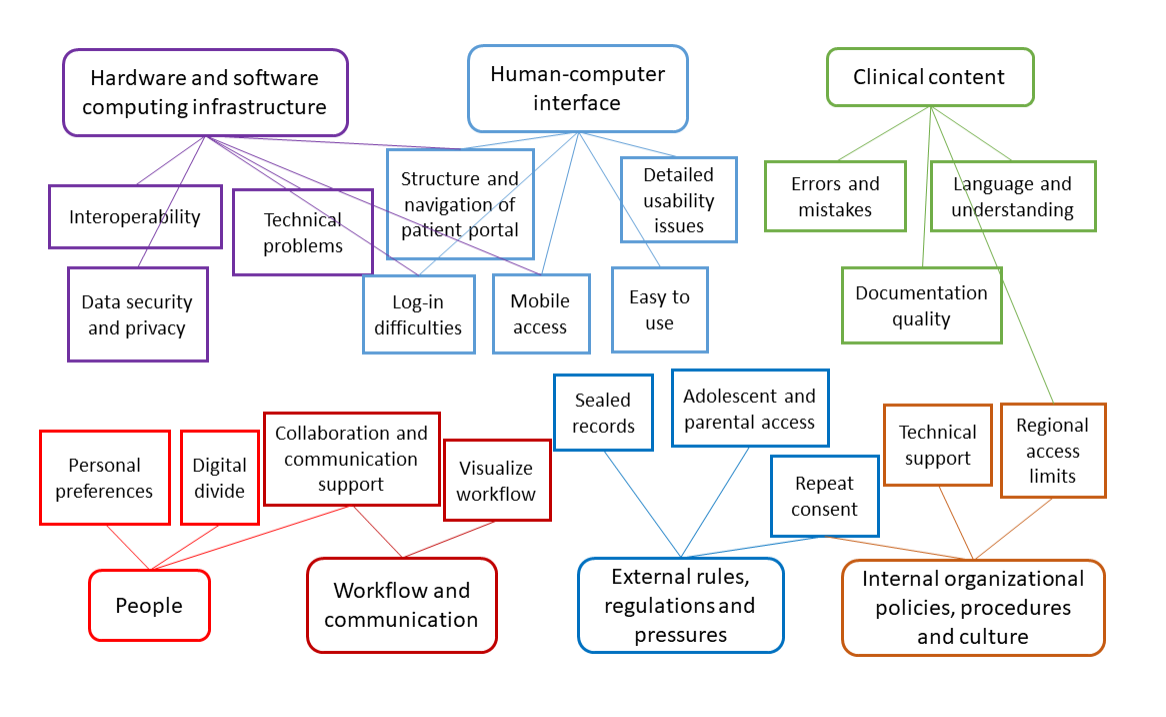
Categories organized according to the sociotechnical framework.

The analysis resulted in 19 categories, which could be grouped under 7 of the 8 dimensions in the sociotechnical framework developed by Sittig and Singh [24] (Figure 1). Some of the categories had links to >1 dimension in the framework, as indicated by the lines in the figure. Although quantification of results is not common in qualitative analysis, we present the number of answers connected to each category in Table 1, as some categories were much more common in the findings than others.

The categories related to the sociotechnical domains are further described in the following sections.

**Table 1:** Frequency of categories (N=186).

Sociotechnical domain and category			Frequency, n (%)
**Hardware and software computing infrastructure**			
	Interoperability	3 (1.6)	
	Data security and privacy	2 (1.1)	
	Technical problems	9 (4.8)	
**Clinical content**			
	Errors and mistakes	4 (2.2)	
	Language and understanding	14 (7.5)	
	Documentation quality	3 (1.6)	
**Human-computer interface**			
	Structure and navigation of the patient portal	22 (11.8)	
	Log-in difficulties	6 (3.2)	
	Mobile access	5 (2.7)	
	Easy to use	3 (1.6)	
	Specific usability issues	7 (3.8)	
**People**			
	Personal preferences	3 (1.6)	
	Digital divide	6 (3.2)	
**Workflow and communication**			
	Collaboration and communication support	8 (4.3)	
	Visualize workflow	2 (1.1)	
**Internal organizational policies, procedures, and culture**			
	Regional access limitations	78 (41.9)	
	Technical support	2 (1.1)	
**External rules, regulations, and pressures**			
	Repeat consent	7 (3.8)	
	Adolescent and parental access	2 (1.1)	
	Sealed records	2 (1.1)	

#### Hardware and Software Computing Infrastructure

Three categories were associated with the first domain: interoperability (3/186, 1.6%), data security and privacy (2/186, 1.1%), and technical problems (9/186, 4.8%). Interoperability issues raised by the respondents were mainly related to health care professionals not having access to information from other organizations, and the respondents expressed concern that this was the case:

Synchronize so that different regions can see each other’s records. Now I have to deliver paper copies from one region to the other. Horrible, now that everything is digital.

A few respondents were concerned about how their data were protected and who had access to them:

I think there should be easy to find (short) information describing how my information is protected. It’s funny that my entire record is available online without me even knowing it...is it uploaded when I log in or is it there all the time?

Most comments in this domain were related to technical issues experienced by the respondents. Most described that the system was slow and appeared immature. Several comments also specifically highlighted access problems in a specific region.

#### Clinical Content

Three categories were associated with the clinical content: errors and mistakes (4/186, 2.2%), language and understanding (14/186, 7.5%), and documentation quality (3/186, 1.6%). Errors and mistakes included both positive and negative comments: most agreed that it is good that patients can find errors or mistakes, although some wished for easier ways of correcting such errors:

It is good to check the record from time to time, since there may be errors in the record that I can sort out together with healthcare.

Limited, it is not possible to write comments or change errors.

Approximately 7.5% (14/186) of comments in this category were related to medical language and understanding. Most of the comments related to difficulties in understanding the medical language and suggestions to include explanations of medical terms to facilitate users who do not have a health care background. However, there were also comments that highlighted that a patient can often understand the notes based on the context:

I’m surprised that it’s so easy to understand what the doctors write. At least in my records, I’ve been easily able to follow what has happened and who did what. It increases my trust in healthcare.

Health care professionals made several of the comments, stating that they have no problem understanding the record themselves but that they worry that other patients may find it difficult. Finally, a few respondents complained that health care professionals did not use the correct structure or keywords when documenting or that there were discrepancies between documentation from different medical specialties. This could be considered a type of error or mistake but is expressed more as a form of poor documentation practice.

#### Human-Computer Interaction

Not surprisingly, the human-computer interaction dimension had 5 categories related to it: structure and navigation of the patient portal (22/186, 11.8%), log-in difficulties (6/186, 3.2%), mobile access (5/186, 2.7%), ease of use (3/186, 1.6%), and specific usability issues (7/186, 3.8%).

The structure and navigation of the patient portal was by far the largest category related to this dimension, and the issues described here concerned both general navigation issues (eg, too many clicks to reach Journalen) and challenges in understanding the relationship between different e-services offered on the patient portal:

Once you’re in Journalen, its ok, but you have to understand the underlying structure to find the way...should I pick Journalen or Healthcare Events? Why can’t I access the children’s records from their account in the patient portal?

Some respondents also described challenges in logging into the patient portal. It is not necessary to sign up for portal access; however, one must have an electronic ID (eID) downloaded on their device. Most people in Sweden use a service provided by their bank; therefore, an eID is often referred to as a BankID. The eID can then be used to access several different public or private e-services, for example, to do tax returns, apply for parental or sick leave, or access web-based banking. However, understanding how to first download and install an eID can be challenging:

I would not have been able to get a BankID without help, or to access the patient portal without help. I have told a lot of people about this service, but they can’t access it because they don’t have a BankID.

Several comments were also related to the mobile interface. An increasing number of users access the patient portal using a mobile device (most often a smartphone), and there were complaints that the interface was poorly adapted for mobile access:

I think the user interface is a bit cumbersome and it is not always clear where you can find the information you’re looking for. I usually use my mobile phone and the user interface is poorly adapted for mobile use. Perhaps a special mobile app would be good.

Although most respondents took the opportunity to describe aspects that needed improvement, a few also expressed that the system was easy to use:

Journalen seems more coherent and stable than the 8 healthcare IT-systems I have worked with professionally.

Finally, some respondents gave very specific feedback on usability issues they had encountered, for example, a link that did not work and data that needed to be entered in a specific format. An issue that was raised that could be especially important related to the function of allowing patients to request a prescription renewal. At the time, there was no connection between the form the patient filled in and the medication list; therefore, the patient had to manually enter the information about the prescription, increasing the risk for errors:

It would have been easier if one could choose an old prescription that needed renewal than to type everything again.

#### People

Some of the comments related to personal preferences (3/186, 1.6%) or the barriers to using digital services that exist, and how this type of eHealth service may not be used by many, potentially increasing the digital divide (6/186, 3.2%):

Waste of time, I’d rather discuss directly with my physician.

I’d prefer a paper record instead.

Most of the comments relating to the digital divide naturally voiced concern on behalf of others, as the respondents were obviously able to access Journalen themselves. Comments were related to socioeconomic factors, functional variations, age, and experience of using computers and digital tools:

The people I know that use the service are all middle class—no one from working class. I see a clear difference there.

The service is not accessible to people with certain types of disabilities and can be difficult for them to use.

Older people and those with limited experience of using computers will have a hard time to use this function, for some people it will be impossible. You should organize special information meetings with instructions for patients and their families.

#### Workflow and Communication

The dimension of workflow and communication had 2 categories: collaboration and communication support (8/186, 4.3%) and visualizing workflow (2/186, 1.1%).

Several respondents expressed a wish to use Journalen in a more collaborative way with their health care professionals, for example, by discussing the content in the record:

I wish the doctor took the initiative and discussed the information in the record with me. When I’m in the office with the physician my mind goes blank and it’s difficult to talk about everything you’d like to, you realize after, and it feels like the doctor only gets a fragmented view of who you are. To discuss or add to the record could perhaps give us both a more complete picture.

Several respondents also wanted better tools for communicating directly with health care providers (eg, a chat function or secure emailing).

In one of the regions (Uppsala), users have access to a *log function*, which is a function allowing them to see whoever has accessed their record. Some users commented on how they used this function to see what was happening in their health care process and wanted even more information to keep themselves up to date with what was happening:

In the log, I would like to see the healthcare organization of the person who has accessed my record. Then I might be able to determine whether anything is happening with the referral that was sent, or if someone is checking up on what has happened to me as a patient since I was examined at their clinic.

#### Internal Organizational Policies, Procedures, and Culture

This dimension had only 2 categories, although one of them had by far the most comments: regional access limitations (78/186, 41.9%) and technical support (2/186, 1.1%). The latter had both a complaint and positive feedback.

Issues around regional access limitations mainly reflected that information users were expecting to find was missing. This is, of course, connected to the clinical content dimension as well; however, the reason some regions show more information than others is the regional adaptations of the national regulatory framework for PAEHR.

Most comments were related to certain types of information (most often laboratory results) that were missing from the record:

I want access to lab results, if they’ve taken a blood test e.g. — now I have to ask each doctor to print the results. I’ve brought these printouts to the primary care centre to facilitate communication.

A lot of functions are not available to me, and then they are “in the way” and a cause for disappointment. E.g., I don’t have access to my lab results, which I would have appreciated, but the function is there but I can’t access them. The system could have been simpler and easier if only functions that are active are shown.

When information was perceived as missing in the record, some respondents also expressed concern that the quality of the documentation was poor and that the lack of documentation reduced their trust in health care:

A problem I experience is that healthcare doesn’t seem to document properly in the record. I have a feeling that there is a real record...but I can only see part of it. Or the documentation is so poor that what I see is all there is. If that’s the case, it worries me.

Another common complaint was that information was not retrospectively available. Many regions decided to only make data from the records available on the web from the date they went live with patients’ access, whereas a few regions provided access to data retrospectively:

I thought more than the last month’s data would be in the record...

Most regions also applied restrictions to certain clinical areas (eg, notes made in psychiatric care). A few respondents commented on this specifically, expressing that the lack of information made access less useful and that the specific blocking of mental health records felt discriminating:

I would use Journalen more if the whole record was included. The fact that the psychiatric notes are not included makes me feel discriminated and fragmented as a person. Body and mind affect each other and somatic care needs to consider what happens in psychiatry and vice versa.

Overall, the regional adaptations of the national regulatory framework caused both confusion and frustration, and when information was blocked, the respondents expressed that it reduced the usefulness and usability of the PAEHR.

#### External Rules, Regulations, and Pressures

Finally, a few categories were connected to external rules, regulations, and pressures: sealed records (2/186, 1.1%), adolescent and parental access (2/186, 1.1%), and repeat consent (7/186, 3.8%).

As a patient, one can request to have the whole or parts of their record to be sealed for access, hindering themselves from accessing it on the web or stopping the record from being accessible to other health care professionals involved in their care. This function was rarely used, although a few respondents who had sealed parts of their records expressed concern:

It’s confusing when it says I can’t access my record when I just wanted parts of it to be blocked for other healthcare providers.

The most frequent complaint in this category was related to having to consent to access the record every time the system was accessed. This was a great source of frustration for users; however, the design of the system took strict privacy and web-based access guidelines into account when designing the system:

I have to check a box that I have understood the same information every time I log in, the first time surely would have been enough?!

A few respondents commented on their access to their children’s records. According to national regulations, legal guardians (most often parents) have automatic access to their children’s records until they are aged 13 years. The age limit has been set in dialog with youth health care specialists and teenagers to protect adolescents’ right to privacy. At the time of the survey, personal access to one’s own record was granted only when they were aged 18 years:

It’s bad that you can’t see the children’s records when they turn 13, and they can’t log in themselves until they turn 18! A gap of 5 years without access to the online record!

Since then, the age for personal access has been lowered to 16 years; however, there is still a gap of 3 years between the loss of parental access and the activation of personal access.

## Discussion

### Principal Findings

To summarize, the results indicate that the respondents of the survey, who were also end users of the Swedish PAEHR Journalen, rated it fairly high on the SUS; however, many sociotechnical usability issues were identified through the respondents’ free-text answers. Before discussing the results in more detail, we would like to address some of the methodological limitations of this study.

### The Web-Based Survey

The survey distribution may have created a selection bias in the study, which should be considered when interpreting the results. The survey was distributed through the national patient portal and was only accessible to people who logged in and accessed the PAEHR. This was intentional, as the main aim of the study was to explore the experiences of people who had used the e-service; however, in doing so, we also excluded anyone who had previously struggled with poor usability and chosen not to continue using the service. Thus, users who were most critical to the service or its usability were likely not represented. If we had recruited individuals to represent the entire Swedish population, the results may have been different. Another potential bias was that the service at that time was not fully implemented throughout Sweden, and usability flaws that may occur for users who are patients in various regions may not have been detected. Thus, it is likely that the results would have covered other usability issues if the studied service would have been implemented simultaneously in all regions.

In addition, it was not possible to determine whether the participants of the survey were representative of all users of Journalen. As in most survey studies, the participants formed a small sample of all possible users, and many more users than those who answered the survey logged into Journalen during the 5 months when the survey was open. We do not know whether the survey respondents’ demographic distribution is representative of all users of Journalen, and the subset of respondents included in this qualitative analysis was even smaller. Despite this, we can assume that other users likely experienced the usability issues identified by our respondents as well.

Formal usability evaluations should be performed to complement these results to provide more details on the specific usability flaws encountered by end users and how these could best be addressed. However, capturing the daily frustrations of users related to, for example, limited access to certain types of information can be difficult in a traditional usability laboratory test where real patient data most likely cannot be used. Therefore, we argue that the results presented herein are of great importance.

### Information Access Through a National Solution

The most frequent frustration commented on by the respondents was lack of access to specific information. We related this problem to local regulations, as each health care region is autonomous and thus has the opportunity to choose which information to share [27]. This proved to be not only difficult for end users to understand but also frustrating, potentially causing distrust in health care, as important information seemed to be missing from the record. Since then, a new regulatory framework has been agreed upon in Sweden, which states that all patients should have immediate access to all clinical documentation from tax-funded health care [28]. However, this change in the framework has not been enforced, and we continue to see differences between regions. Clinical notes from psychiatric care have, for example, often been excluded from patient access, not only in Sweden [29,30]. As our results indicated, excluding specific parts of the documentation can increase the sense of stigmatization and distrust. However, over the years, progress has been made, and when it comes to, for example, notes from psychiatric care, currently, 17 of 21 Swedish regions provide patients access [22] compared with only 2 when the survey was conducted [21].

### PAEHRs as a Tool for Collaboration

An important aspect raised by some respondents was the need for more collaborative functions in the PAEHR. Using record access as a means of initiating and supporting dialog with their health care team was a wish expressed by several respondents. However, it appeared that the record was not used in this way by health care professionals [17]. When asked specifically about whether they as patients discussed the record content with health care professionals, 30.79% (766/2488) of the respondents completely or partly agreed, whereas 51.21% (1274/2488) did not agree or not at all agree. There still appears to be an underused opportunity to use the PAEHR for collaboration [31,32], especially considering that from the patient perspective, improved communication with health care professionals was stated as one of the most important reasons for accessing the PAEHR [17].

### PAEHRs and the Digital Divide

Although we only reached actual users of the PAEHR Journalen through this survey, some comments were related to difficulties in logging in and using an eID. Some participants stated that they would not have been able to access the PAEHR without help, and others reported that friends and relatives were unable to access the PAEHR as they did not have an eID. Low digital literacy is a well-known barrier to accessing and using eHealth [33] and is often correlated with older age or low education and socioeconomic status. Although we cannot draw any conclusions regarding this from our survey, the respondents in this study also had a higher education level than the general population. Although older users may experience more difficulties accessing and using the PAEHR, they also reported the greatest benefit of doing so [34]. Today, there is still very limited education or introduction of the PAEHR Journalen to patients; rather, they are expected to be able to use and understand the system intuitively. In fact, when asked whether they had been informed by health care professionals about the existence of Journalen, only 13.49% (335/2483) of the respondents agreed, and only 8.19% (203/2480) had been encouraged by health care professionals to read their records [17]. Although this may work well for many, making further educational resources available to patients may be a way of improving patients’ experiences of using Journalen and could alleviate some of the problems described by the respondents in this study.

Since the survey was launched in 2016, the use of the Swedish PAEHR Journalen has increased rapidly, going from 77,000 individual users per month in January 2016 to 1,850,000 in January 2022 [25]. Uptake has been slow but steady, and the COVID-19 pandemic boosted use during 2020 and 2021 when, for example, COVID-19 test results were accessible through the PAEHR in most regions. We can assume that more people currently use both eIDs and the national patient portal, and a repeat survey is planned to take place in 2022, where we expect to reach a more diverse group of respondents.

### PAEHRs and Proxy Access

Several respondents brought up proxy access, focusing specifically on parents’ access to their children’s records. The current regulation in Sweden automatically provides legal guardians (most often parents) access to a child’s record until they are aged 13 years. The teenagers themselves can log in and access their records at the age of 16 years, leaving a 3-year gap when neither the child nor their parents have access to the record. This was critiqued by several respondents, as was the difficulty in finding the child’s record in the patient portal. So far, limited research into the specific area of parental proxy and adolescent access has been performed in Sweden, and we know that regulations in this area differ between countries [2].

When it came to proxy access for other patient groups, there was a function for sharing parts of or the entire record with another person of the patient’s own choosing at the time of the survey. However, this function was not widely used by the survey respondents [35], potentially as they managed their own health themselves. In 2018, the functionality was removed from the PAEHR as it was considered noncomplying with current legislation on the handling of patient data. A revision of the legislation is currently underway, and functionality may be reopened in 2023. Further research into proxy access (both parental and other) is needed.

### PAEHRs as a Tool for Patient Safety

Patient safety is often discussed in relation to patient portals and record access, with varying opinions on whether the effect is positive or negative. When patients are given web-based access to their records, it is not uncommon for them to discover errors or mistakes [36,37]. This was also commented upon by the respondents, where some used their access to ensure that no misunderstandings had occurred. However, the means for correcting errors were missing from the PAEHR, which was considered a problem by some respondents. Interesting research in the United States, where patients are provided with feedback tools to highlight and potentially correct errors, indicates that there could be real patient safety benefits from implementing such tools [38], something to be considered in other contexts as well.

Misunderstandings and confusion from accessing the records have been a concern for many who worry that patients may come to harm [39,40]. In this study, several respondents stated that the record could be difficult to understand and suggested, for example, that links to terminologies or explanations could be automatically added to the text. However, others also expressed that the records were surprisingly easy to understand. There can be several reasons why patients’ experiences differ here: different levels of health literacy may affect their understanding, different medical specialties may use more or less difficult terminology, and individual health care professionals may express themselves in more or less easy-to-follow notes. Regardless, there appears to be room for improvement in this area, and both patient and health care professional education may be needed. How patient access may affect the way health care professionals actually document information is also an underexplored area [41], especially in domains such as mental health, where health care professionals have reported leaving important information out of the record as patients gain access to it [42,43].

### Conclusions

Although the respondents of the survey regarding the PAEHR Journalen were overall satisfied with its usability [21], they also experienced important challenges when accessing their records. For all patients to be able to reap the benefits of record access, it is essential to understand both the usability challenges they encounter and, more broadly, how policies, regulations, and technical implementation decisions affect the usefulness of record access. The results presented here are specific to the Swedish PAEHR Journalen but also provide important insights into how the design and implementation of record access can be improved in any context.

## Abbreviations

EHR: electronic health record
PAEHR: patient-accessible electronic health record
SUS: System Usability Scale
eID: electronic ID
